# Neurodevelopment and Cognitive Impairment in Parents and Progeny of Perinatal Dietary Protein Deficiency Models

**DOI:** 10.3389/fnins.2019.00826

**Published:** 2019-08-21

**Authors:** Nosarieme O. Abey, Osaretin A. T. Ebuehi, Ngozi O. A. Imaga

**Affiliations:** Department of Biochemistry, Faculty of Basic Medical Sciences, College of Medicine, University of Lagos, Lagos, Nigeria

**Keywords:** neurobehavior, cognitive function, protein deficiency, neurotransmitter, transgeneration, perinatal

## Abstract

There is an absolute dependence of the concept of development on supply of adequately balanced nutrients especially during the perinatal age which is critical to development. Therefore, an upgraded nutrition is specially required during gestation and lactation, as this is the critical period of neurodevelopment. This study sought to investigate the effect of protein deficiency during gestation (F_0_) and lactation through to adolescence on neurological functions of subsequent (F_1_ and F_2_) generations, establishing the possible consequential mechanistic association. Rats in four groups were fed different rations of protein diets (PD) as formulated: 21% PD, 10% PD, 5% PD and control diet (standard rat chow, containing 16–18% protein), from adolescent through to gestation and lactation, next generations were weaned to the maternal diet group. Neurobehavioral studies (which include; Surface righting reflex, Negative geotaxis, Learning and Memory tests), brain oxidative stress and quantification of serotonin and dopamine levels in the brain were conducted. Result shows significantly altered neurobehavior, reflected in the reduction of reflex response and postural reaction score at *P* ≤ 0.05. There was also a transgenerational cognitive impairment of brain function in the F-generations, following perinatal protein malnutrition as shown in the Y-maze result, measuring spatial memory and Morris water maze result (cognition), providing a background for the observed sensorimotor response. The significant increase in dopamine level, decrease in the antioxidant capacity of the protein deficient brain groups are consistent with significantly altered serotonin system, critical to neurodevelopment and functional activities of learning and memory. Therefore, persistent early life protein deficiency mediates dysfunction in neurodevelopment and this involves life-long changes in key neurotransmitters and the brain redox status underlying the neurobehavioral display.

## Introduction

Epidemiological studies have indicated that perinatal age exposures; including diet and nutrition affects directly later life developments, due to a shift or programed conditions. One of the well reported consequences are highly induced integrated responses in the endocrine homeostasis. which results in later life to subsequent and persistent changes in developmental trajectory yielding an altered phenotype which may appear as a physiological defect (Chan et al., 2011). Evidence of the dependence of brain development on nutrition is increasingly gaining interest. Nutritional status has been demonstrated to be associated with postnatal brain growth and maturation, subsequently impacting the neurodevelopment which may persist to adolescence (Black, 2018). Nutrition could also potentially protect against injury (Hurley et al., 2016). During the critical period of brain development (0–2 years), i.e., the first 1,000 days, brain development is rapid, with nutrition playing an important role as one of the environmental influences on the coding and expression of the genetic code for structural and functional impact. Subsequent cognitive and emotional process coupled with the entire brain development in animal and human subjects have recently illustrated that the duration of exposure, chronicity, and severity of nutritional deficiencies have differential effects on brain development (Colombo et al., 2018). Fetal and neonatal programing have been reported to be the underlying mechanism for the far-reaching adverse consequences in humans following inadequate protein intake during gestation and postnatal periods (Wu, 2016). This risk factor results in not only impaired growth of fetuses and infants, but also some other problems such as metabolic syndrome (including hypertension, obesity, and diabetes) and low quality of life as adults (Sosa-Larios et al., 2017). There have been quite a few focuses on unveiling nutraceutical significance in neurodevelopment, which has contributed immensely to disability in individuals who bear the burden. In developing/low-resource settings it may be associated with a significant risk of impoverishing the health reserves which threatens survival and deepens poverty. This study sought to investigate the effect of maternal protein deficiency during gestation (F_0_) and lactation on neurological functions of subsequent (F_1_ and F_2_) generations, establishing the possible consequential mechanistic association.

## Materials and Methods

### Ethics Statement

This study was carried out in accordance with the guidelines of health research Act 2004, for standard care and use of laboratory animal models. Ethical approval was given by the Health Research Ethics Committee (HREC) of College of Medicine University of Lagos (REC 11), Nigeria.

**FIGURE 1:**
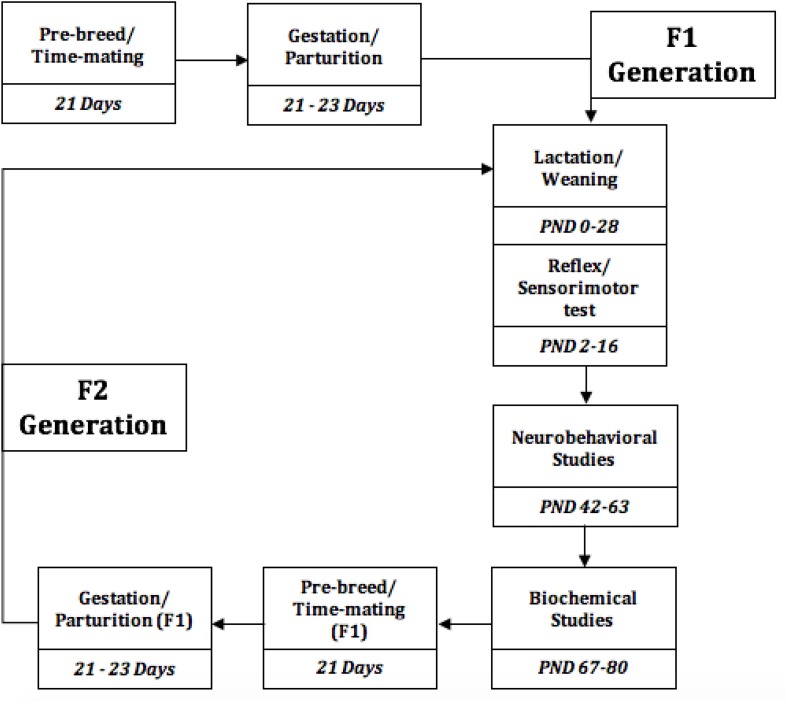
Experimental Timeline of the events, from the study initiation to termination. PND, Postnatal days.

Forty virgin female Sprague Dawley rats (aged 6–8 weeks) were obtained from the animal house of the College of Medicine University of Lagos, Lagos Nigeria (Figure 1). Following the ethics of the standard care of animal models in research, rats were kept in groups in clean and capacious plastic cages (seven per cage) under standard laboratory conditions including well aerated room, good lighting, with suitable temperature (30° ± 2°C) in a neat environment and at a 12-h light/dark cycle. The animals were divided into four (4) groups and acclimatized for 2 weeks, where they had access to standard rat chow and water *ad libitum*.

### Grouping

The grouping was based on the diet received by the rat. Grouped rats received *ad libitum* formulated diets which include either of these diet formulations: 21% PD (protein diet), 10% PDD (Protein deficient diet 1), 5% PDD (Protein deficient diet 2), and CP (standard rat chow, containing 16–18% protein) throughout gestation and lactation, forming Groups 1 2, 3 and 4, respectively, and pups were weaned to maternal diet.

### Diet Formulation

The varying protein quality diet for rat was formulated using local materials such as: Maize, SBM, GNC, Fish meal cassava starch, oil etc., all in varying percentages toward the attainment of the desired percentage of protein in the diet. This feed was formulated by the help of some experts at federal institute of industrial research Oshodi (FIIRO). Formulated feeds were given to the rats.

### Prenatal Treatment

Vaginal cytology was evaluated 3 weeks pre-breed period, and the rats were time-mated with a certified reproductive male in each treatment group, the confirmed pregnant rats were separated to produce F_1_ generation, this process was repeated for F_1_ generation to produce F_2_ generation, while the different diet group feeding continues. Presence of spermatozoa in vagina smear confirms day 1 of pregnancy. Weaning was done at postnatal day 21–28.

### Reflex and Sensorimotor Test

#### Surface Righting Reflex (SRR)

The test was evaluated on postnatal day 2 through to 6. This test was carried out to measure the motor function and coordination by placing pups in a supine position and the time taken to adopt normal position was recorded, scored within cut-off time of 0–3 s (Naik et al., 2015).

#### Negative Geotaxis Reflex

The vestibular and proprioceptive functions were evaluated in rats (postnatal day 2–14, every other day) were placed head down on an inclined board (about 45°), the time it took the animal to show a postural reaction by turning upright normally was recorded and scaled to score (Naik et al., 2015).

### Neurobehavioral Tests

At six (6 weeks), offspring were randomly selected (*n* = 5) for different neurobehavioral trainings and trials.

#### Y-Maze

The test was conducted as a modified method described by Yarube et al. (2016), The number and the sequence of arms entered were also recorded. The parameters were activity, defined as the number of arms entered, and percent alternation, calculated as the number of alternations or triads.

#### Moriz Water Maze

A wide plastic cylindrical tank of about 120 cm width, that is surrounded by a wall (45 cm) and filled with water that was made opaque to reduce light penetration (25°C) was set up for the experiment. A rescue platform, 10 cm high was hidden at the center of the tank, below water level. Rats were given three swimming sessions on each of five consecutive days. At trial, the time taken to locate the platform (escape latency) was measured (Wang et al., 2017).

### Serotonin and Dopamine Quantification

The brain serotonin and dopamine concentration in the rat were determined using high performance liquid chromatography (HPLC), Agilent 100 series with VWD detector degasser, Quat Pump, Col Com and a manual injector system. Striatum tissues in the brain were collected, homogenized, deproteinized by chilled acetonitrile and filtered using syringe filter in preparation for injection. Serotonin and dopamine contents were determined by comparing the peak height ratios of the standards (5-HT and dopamine) used as calibrators and the chromatogram of unknown (Ebuehi and Abey, 2016).

### Brain Antioxidant Assays

The brain antioxidant capacity was measured using the following biomarkers.

#### Determination of Reduced Glutathione (GSH)

The reduced glutathione level was determined based on the yellow color formed after reacting with 5,5’dithiobis- 2-nitrobenzoic acid (DTNB), which is then read at 412 nm (Ellman, 1978).

#### Determination of Catalase Activity (CAT)

The activity of the enzyme catalase was analyzed by measuring the initial rate of hydrogen peroxide (50 mM) decomposition, where one unit is the amount of enzyme that hydrolyses 1 mol. of H_2_O_2_ per minute, following method described by Cohen et al. (1996).

#### Determination of Super Oxide Dismutase Activity

The super oxide dismutase (SOD) activity was determined according to the modified method of Sun and Zigman (1978), determined by the ability of SOD to inhibit the autoxidation of epinephrine, taken as the difference between superoxide anion production and decomposition, measured by the increase in absorbance at 320 nm.

#### Determination of Lipid Peroxidation Malondialdehyde (MDA)

Malondialdehyde is red specie absorbing at 535 nm (Buege and Aust, 1978). It is formed from the breakdown of polyunsaturated fatty acids, serving as a convenient index for determining the extent of lipid peroxidation that reacts with thiobarbituric acid. Absorbance taken at 535 nm, corresponds to the concentration of MDA per mg protein.

### Determination of Concentration of Nitric Oxide

Nitric Oxide was assayed by the Griess reaction method (Bryan and Grisham, 2007). 0.2 ml of the Griess reagent mixed with 3 ml of the sample and incubated at room temperature for 10 min. Absorbance was read using the spectrophotometer at 540 nm. This procedure is not stoichiometric and thus, a standard curve of sodium nitrite was plotted in order to extrapolate the values for unknown samples.

#### Statistical Analysis

All data were analyzed with maternal and weaning diets as factors. Results are presented as the mean ± SEM. Statistical analysis was performed using GraphPad Prism 7.0 for analysis of variance (ANOVA) followed by *post hoc* Turkey’s test, when appropriate; *P* ≤ 0.05 is considered significant.

## Results

**FIGURE 2:**
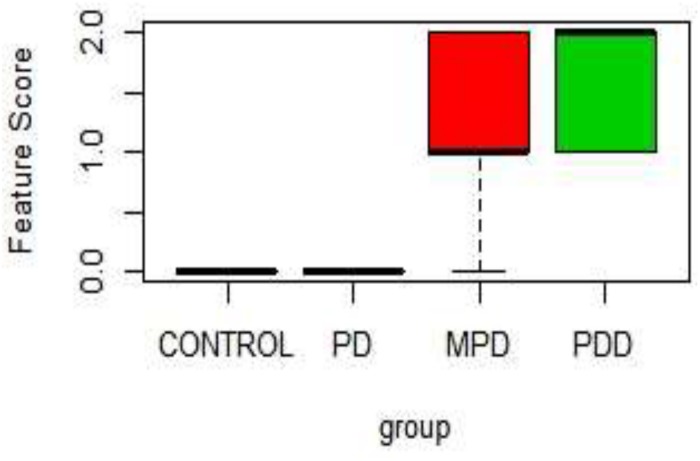
Boxplot of Physical characteristic scores in the different diet group. PDD: 5% Protein Diet, Mild PD: 10% Protein Diet, PD: 21% Protein Diet, and CONTROL. Score Keys – Scale: 0–2: 0, No characteristic display; 1, Mild characteristics display; 2, Prominent Characteristics display. Features Scored: Paleness, Muscle wasting, Stunted growth, Alopecia, Edema, and lesion.

Table 1 shows the score on a scale of 0–2 based on prominence/severity of characteristic displayed. Control and 21%PD had no display of the scored features, this relationship is represented on the box-plot (Figure 2).

**TABLE 1:** Physical features of rats fed different protein diets in the F_1_-generation.

**Characteristics**	**Control**	**21% PD**	**10% PD**	**5% PD**
Paleness	0	0	0	2
Muscle wasting	0	0	1	2
Stunted growth	0	0	1	2
Alopecia (hair thinning and loss)	0	0	1	2
Edema	0	0	2	1
Lesion	0	0	2	1

*Features Scored: Paleness, Muscle wasting, Stunted growth, Alopecia, Edema, and lesion. Score Keys – Scale: 0–2: 0, No characteristic display; 1, Mild characteristics display; 2, Prominent Characteristics display.*

In Figure 2, the 5% PD and 10% PD had characteristic score skewed toward the positive, reflecting a higher display, while control and 21% PD skewed toward the negative, with no visible display. These distinctive features of perinatal protein deficiency compared to control and 21% protein diet group include; prominent paleness, muscle wasting, hair loss and stunted growth.

**FIGURE 3:**
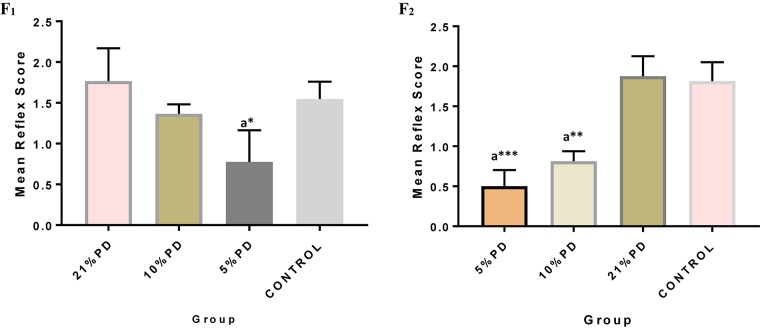
Mean reflex response score in each of the diet groups of rats of F_1_ and F_2_-generations. Data are expressed as mean ± SEM, Control (standard rat chow containing 16–18% Protein), 21% PD (Upgraded daily recommended intake), 5% (Protein deficient diet), and 10% (Mild protein deficient diet). ^a^significantly different from 21% diet group and or control. ^∗^*P* < 0.05, ^∗∗^*P* < 0.001, ^∗∗∗^*P* < 0.0001.

**FIGURE 4:**
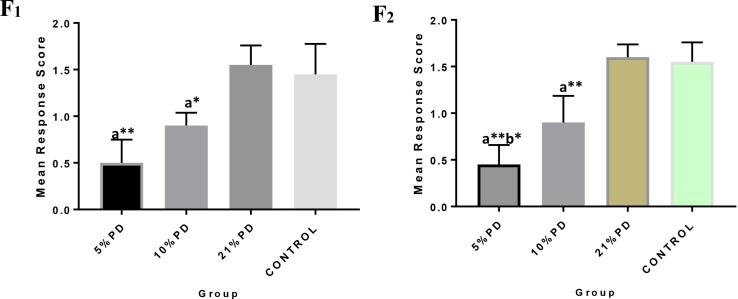
F_1_ and F_2_ Mean response score of the postural reaction in each of the diet group of rats. Data are expressed as mean ± SEM, Control (standard rat chow containing 16–18% Protein), 21% PD (upgraded daily recommended intake), 5% (Protein deficient diet), and 10% (Mild protein deficient diet). ^a^significantly different from 21% diet group and or control. ^b^significantly different from the 10% diet group. ^∗^*P* < 0.05, ^∗∗^*P* < 0.001.

**FIGURE 5:**
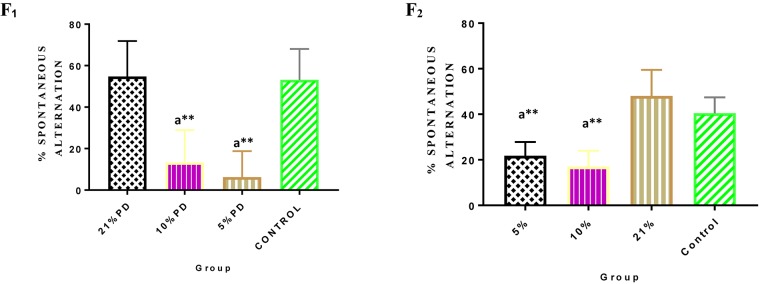
Y-maze Percentage Spontaneous Alternation in each of the diet group of F_1_ and F_2_-generation rats. Data are expressed as mean ± SEM. Statistically significant difference between individual group and the hypothetical value of 50% at *P* < 0.05. $\mathrm{Spontaneous}\,\mathrm{alteration}\%=\frac{\#\,s\,p\,o\,n\,t\,a\,n\,u\,o\,s\,a\,l\,t\,e\,r\,n\,a\,t\,i\,o\,n}{t\,o\,t\,a\,l\,n\,u\,m\,b\,e\,r\,o\,f\,a\,r\,m\,e\,n\,t\,r\,i\,e\,s-2}\times 100$ Control (standard rat chow containing 16–18% Protein), 21% PD (Upgraded daily recommended intake), 5% (Protein deficient diet), and 10% (Mild protein deficient diet). ^a^significantly different from 21% diet group and or control. ^∗∗^*P* < 0.001.

**FIGURE 6:**
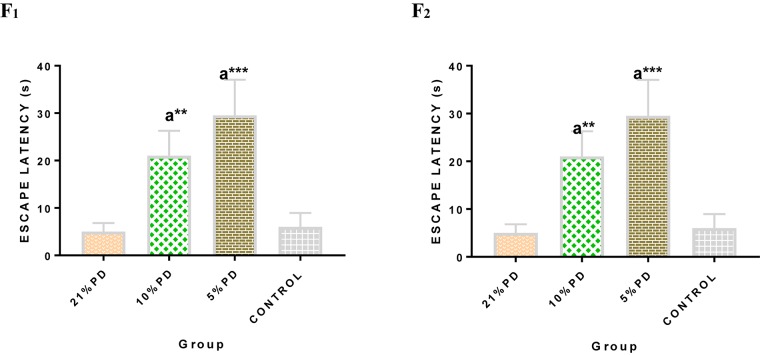
Mean Moriz Water Maze Experiment showing the Escape Latency demonstrated by each group in F_2_-generations. Data are expressed as mean ± SEM, Control (standard rat chow containing 16–18% Protein), 21% PD (upgraded daily recommended intake), 5% (Protein deficient diet), and 10% (Mild protein deficient diet). ^a^significantly different from 21% diet group and or control. ^∗∗^*P* < 0.001, ^∗∗∗^*P* < 0.0001.

Significantly higher response time in the protein deficient models (Figure 3) as well as a negative postural score (Figure 4) in the negative geotaxis test at the early postnatal days of the protein deficient diet groups. In Figure 5, Y-maze% spontaneous alternation result shows significant deficit in the 5% protein diet group in the two (2) generations (F_1_ and F_2_) while 10% was only significant in F_1_-generation. In addition, escape latency observed in Figure 6 shows that the malnourished groups spent longer time to locate the rescue platform during trial sessions, a display of cognitive deficit. With significant dietary factor influence, at *P* < 0.001.

**FIGURE 7:**
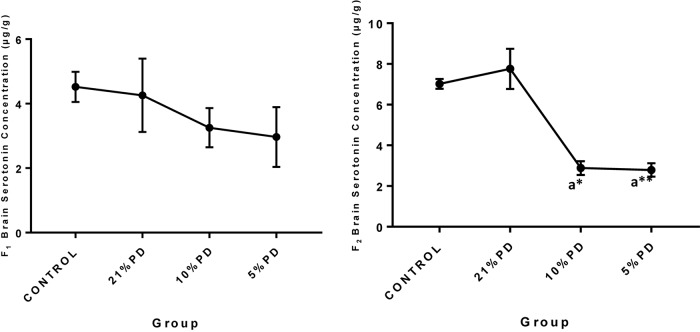
Concentration of Brain Serotonin (μg/g) in the F_1_ and F_2_-generations of rat in each of the different diet groups. Data are expressed as mean ± SEM, Control (standard rat chow containing 16–18% Protein), 21% PD (upgraded daily recommended intake), 5% (Protein deficient diet), 10% (Mild protein deficient diet). ^a^significantly different from 21% diet group and or control. ^∗^*P* < 0.05, ^∗∗^*P* < 0.001.

**FIGURE 8:**
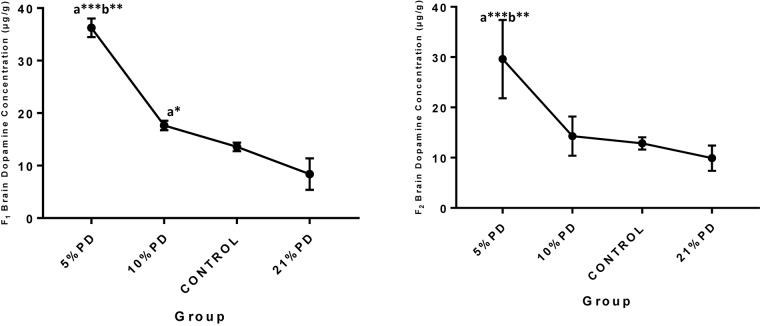
Concentrations of Brain Dopamine (μg/g) in the F_1_ and F_2_-generations in each of the different diet group of rats. Data are expressed as mean ± SEM, Control (standard rat chow containing 16–18% Protein), 21% PD (upgraded daily recommended intake), 5% (Protein deficient diet), 10% (Mild protein deficient diet). ^a^significantly different from 21% diet group and or control. ^b^significantly different from the 10% diet group. ^∗^*P* < 0.05, ^∗∗^*P* < 0.001, ^∗∗∗^*P* < 0.0001.

Serotoninergic and dopaminergic neurotransmission, important in neurobehavioral control and cognition was perturbed in the nutritionally stressed models (Figures 7, 8) which persists to subsequent generation. Endogenous antioxidant status of the malnourished groups were suppressed. Tables 2 and 3, with significant increase in the Malondialdehyde level.

**TABLE 2:** Oxidative stress biomarkers in different diet groups of F_1_-generation of rat models.

**Group**	**Total protein (mg/ml)**	**Catalase (U/L/min)**	**GSH (μmol/L)**	**SOD (U/L/min)**	**MDA (μmol/L)**	**Nitric oxide (mg/ml)**
Control	31.43 ± 6.10	20.72 ± 4.00	45 ± 3.80	0.0249 ± 0.01	0.29 ± 0.09	6.989 ± 1.20
21% PD	41.35 ± 8.00	15.92 ± 3.99	53.14 ± 4.00	0.0438 ± 0.01	1.02 ± 0.9	7.147 ± 0.40
10% PD	34.16 ± 1.41	21.29 ± 1.96	45.89 ± 0.98	0.1989 ± 0.03	2.5 ± 0.7^a**^	1.585 ± 0.70^a**b**^
5% PD	20.23 ± 2.98^a*^	34.89 ± 5.00^a**b*c*^	37.74 ± 4.70^a*^	0.00334^a**^ ± 0.0	24.55 ± 4.6^a**b**^	1.6 ± 1.10^a**b**^

*Data are expressed as mean ± SEM of F_1_ antioxidant status significantly difference from 21% Protein and control Diet group. Control (standard rat chow containing 16–18% Protein), 21% PD (upgraded daily recommended intake), 5% (Protein deficient diet), and 10% (Mild protein deficient diet). ^*a*^significantly different from 21% diet group and or control. ^*b*^significantly different from the 10% diet group. ^∗^*P* < 0.05, ^∗∗^*P* < 0.001.*

**TABLE 3:** Oxidative stress biomarkers in different diet groups of F_2_-generation of rat models.

**Group**	**Total protein (mg/ml)**	**Catalase (U/L/min)**	**GSH (μmol/L)**	**SOD (U/L/min)**	**MDA (μmol/L)**	**Nitric oxide (mg/ml)**
Control	1.9166 ± 0.19	5.9259 ± 0.40	13.051 ± 1.5	0.0116 ± 0.002	0.0673 ± 0.01	9.40 ± 0.94
21% PD	1.965 ± 0.27	8.3951 ± 0.56	16.219 ± 1.3	0.0261 ± 0.001	0.0289 ± 0.01	11.82 ± 0.09
10% PD	1.0224 ± 0.07^a*b⁣*^	60.25 ± 6.90^a⁣*⁣*b⁣**^	11.46024 ± 1.1	0.050435^b⁣*^±0.001	0.279 ± 0.1^a⁣*⁣*b⁣**^	8.08 ± 1.34
5% PD	0.878 ± 0.01^a*b⁣*^	75.062a^**b**^	9.065 ± 0.7^a⁣*^	0.0809 ± 0.0001^a⁣*⁣*b⁣*⁣*c⁣*^	0.356 ± 0.04^a⁣*⁣*b⁣**^	3.264 ± 1.0^a⁣*⁣*b*c⁣*^

*Data are expressed as mean ± SEM of F_2_ antioxidant status significantly difference from 21% Protein and control Diet group. Control (standard rat chow containing 16–18% Protein), 21% PD (upgraded daily recommended intake), 5% (Protein deficient diet), and 10 (Mild protein deficient diet). ^*a*^significantly different from 21% diet group and or control. ^*b*^significantly different from the 10% diet group. ^∗^*P* < 0.05, ^∗∗^*P* < 0.001.*

## Discussion

Neurodevelopment is peculiar to early life and this stage is critically dependent on maternal protein intake. Dietary protein deficiency and its corresponding effect on neurodevelopment critical to survival and quality of life that subsequently follows, perturbs the neurotransmission system and cognition programing. The brain in an attempt to compensate for the nutritional inadequacies, may change its functional activation, as an important signature for the reorganization, providing explanation for the various chemical alterations and behavioral display reported in the result.

As earlier stated by Guo and Katta (2017), protein is crucial to the health of hair follicles, and hence the alopecia observed in the protein deficient groups, also in accordance with the previous research (Belluscio et al., 2014), stunted growth is attributed to the suboptimal intrauterine condition of the nutritionally challenged group.

Protein deficiency caused perturbations in brain development observed as delayed neurological reflex and postural response. Therefore, the neuronal response of these deficient models has been negatively impacted leading to the weakening of the nerves and deterring communications and potentiation essential for learning (Naik et al., 2015). The working memory and cognition of the protein deficient group lagged significantly; taking more time to perform learned task, this suggests a fundamental defect in the neurodevelopmental process and degeneration in brain function, which persists in both parent and progeny.

Malnutrition in early life as earlier reported (Chertoff, 2015), affects the neurochemistry and morphology of the hippocampal structure, which is known to be involved in learning and memory. One of the key factors on which brain function depends is the availability of neurotransmitter molecule for release into synapse, this alteration in the neurotransmitter scale may therefore underlie the functional deficit displayed by the protein malnourished groups. In addition, dopamine alteration perturbs neural encoding, this suggests a clinical association with other dysfunctions such as anxiety and psychosis (Ebuehi et al., 2001, 2009; Kelly et al., 2017). In malnutrition condition, free radicals mediate tissue damage, due to the inadequate protective and repair mechanisms in the protein-deficient models (Feoli et al., 2006). Therefore, maternal undernutrition may contribute to the transgenerational imbalance in the brain redox status of the following generation. The developmental behavior, and cognitive deficits observed on perinatal malnourished models might derive their effects from mechanisms which include the perturbed redox status and neurotransmission as an insult to the Brain.

## Conclusion

Perinatal protein deficiency altered the brain development in parent and offspring. Increased oxidative damage and altered neurotransmission in the brain of F_1_ generation of rats, underlie the perturbed neurobehavioral display and cognitive deficit in the nutritionally challenged groups. Therefore, persistent perinatal exposure to protein malnutrition increases the risk of cognitive defect and other brain disorders in subsequent generations. This study affirms the importance of dietary protein at the early developmental life process laying emphasis on the mechanism associated with the resultant transgenerational neurodevelopment and cognitive impairment following persistent perinatal protein deficiency. More work needs to be done to establish a molecular mechanism for these effects which could be linked to neurodegenerative disorders.

## Data Availability

The datasets generated for this study are available on request to the corresponding author.

## Author Contributions

NA, OE, and NI designed the study. OE and NI constructively supervised the analyses of the study. NA managed the analyses, statistical analysis, the literature search, and wrote the first draft of the manuscript. All authors read and approved the final manuscript.

## Conflict of Interest Statement

The authors declare that the research was conducted in the absence of any commercial or financial relationships that could be construed as a potential conflict of interest.
